# Neurotoxicity of Polycyclic Aromatic Hydrocarbons: A Systematic Mapping and Review of Neuropathological Mechanisms

**DOI:** 10.3390/toxics10080417

**Published:** 2022-07-25

**Authors:** Tosin A. Olasehinde, Ademola O. Olaniran

**Affiliations:** 1 Nutrition and Toxicology Division, Food Technology Department, Federal Institute of Industrial Research Oshodi, Lagos 102215, Nigeria; 2 Discipline of Microbiology, School of Life Sciences, Westville Campus, University of Kwazulu-Natal, Durban 4000, South Africa; olanirana@ukzn.ac.za

**Keywords:** polycyclic aromatic hydrocarbons, neurotoxicity, neurodegeneration, memory function, neurological disorders, oxidative stress, cholinergic dysfunction

## Abstract

Several studies present the neurotoxic effects of polycyclic aromatic hydrocarbons (PAHs), a class of environmental pollutants capable of causing neurological deficits. However, a collective review approach to this research topic is scarce. This study presents the effect of PAHs on the central nervous system using a bibliometric approach. The neuropathological mechanisms of PAHs are also highlighted. Published articles were searched for in the Scopus and Web of Science databases from January 1979 to December 2020 using the keywords ‘polycyclic aromatic hydrocarbons’ and ‘neurotoxicity’. The total number of documents retrieved from both databases was 338. Duplicated documents (80) were excluded and 258 articles were used for the final analysis. Our findings revealed that there has been a significant increase in research outputs on this topic in the last ten years. The countries with the highest scientific productivity in this area are USA, China, France and Italy. The result also showed that, in the past few years, global scientific output in research relating to PAH neurotoxicity focused on neurodegeneration, cholinergic function, neurodevelopmental toxicity, behavioural studies, oxidative stress, neuroprotection and therapeutic intervention using different experimental models, including zebrafish, neuronal cell lines, *Caenorhabditis elegans* and rats. Recent studies also revealed the neuroprotective roles of some natural products against PAH-induced neurotoxicity. However, more investigation involving clinical trials is required to emphasize the observed neurotoxic effects.

## 1. Introduction

Polycyclic aromatic hydrocarbons (PAHs) are ubiquitous chemicals and one of the major classes of persistent organic pollutants found mainly in the environment [1]. PAHs are semi-volatile, lipophilic and commonly found in the atmosphere, soils, seawater, vegetation, inland and sediments [2]. They are easily transported within the environment with the possibility of moving from the atmosphere to the Earth’s surface in repeated cycles [3]. Some PAHs that have been identified in sediments, seas and vegetation include benzo[a]pyrene, anthracene, benz[a]anthrancene, fluoranthene, fluorene, pyrene, naphthalene and Chrysene [3,4,5,6]. PAHs are primarily released into the environment due to natural factors through the incomplete combustion of fossil fuels, wood and petroleum products [7]. Human and industrial activities, including the combustion of refuse and natural gas, tobacco smoking, vehicle exhaust, production of coke and coal tar and emission from industrial power generators and incinerators, may also contribute to the release of PAHs into the environment [2,8]. PAHs in the environment find their way into humans via food and dietary sources, soil, drinking water, etc.

Exposure to PAHs has been linked with reproductive toxicity [9], carcinogenicity [10], genotoxicity [11], immunotoxicity [12], teratogenicity [13], systemic inflammation [14,15] and endocrine-disrupting effects [16]. Benzo[a]pyrene is commonly used as a model for PAH-induced toxicity, and utero exposure to this PAH has been shown to cause testicular toxicity, immunosuppression and initiation of tumour formation [17,18]. Moreover, the neurotoxic effect of PAHs has also been established. Studies have shown that PAHs can induce memory impairment, cognitive dysfunction and behavioural problems [19,20,21,22]. Furthermore, short-term and long-term exposure to PAHs has also been associated with neurological deficits [23,24]. Studies on prenatal exposure to PAHs revealed that cognitive impairment and behavioural problems might occur later in childhood [25,26,27]. Furthermore, neurodevelopmental toxicity triggered by some PAHs has been observed in animal models and clinical trials [28,29,30]. This study presents a bibliometric analysis of the global research outputs related to neurotoxic effects of PAHs. This study is essential as bibliometric analysis on this topic is very scarce. The analysis was carried out to investigate the research trends associated with PAH-induced neurotoxicity and to provide insights into the mechanisms of action and new therapeutic intervention studies. Articles related to PAH-induced neurotoxicity published in journals indexed in Scopus and Web of Science in 1979–2020 were used for this study. The searched results revealed the countries with the highest number of research outputs, citations and strong collaborations. Furthermore, the pathological mechanisms of some PAHs and research outcomes of therapeutic interventions that have been reported are highlighted, and possible future research trends and perspectives needed for exploration are presented.

## 2. Methodology

### 2.1. Acquisition of Data

**Figure 1:**
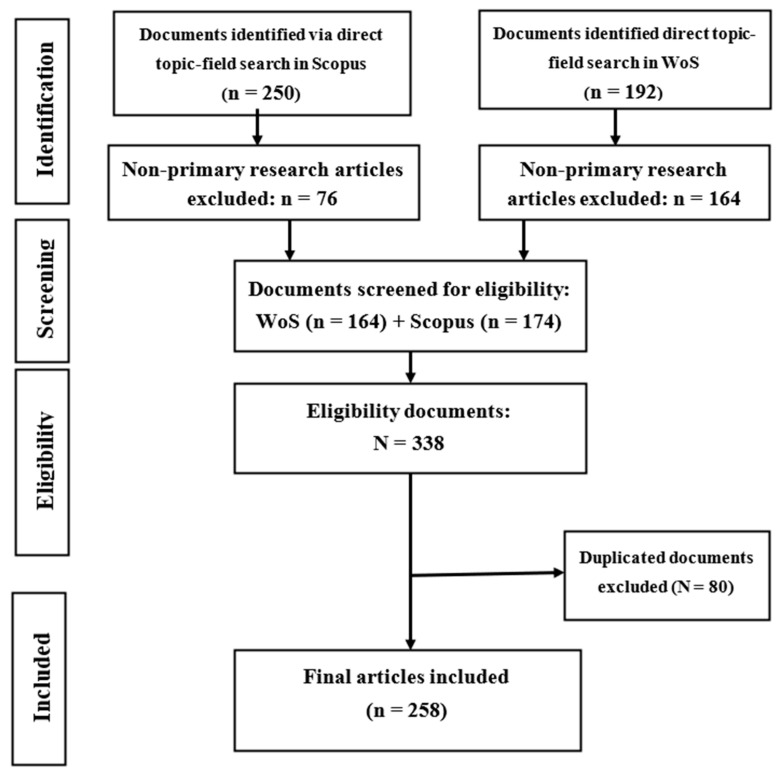
PRISMA diagram revealing article selection and exclusion for final analysis.

The “Preferred Reporting Items for Systematic Reviews and Meta-Analyses” guidelines were used in this study [31]. Scopus and Web of Science databases were used to search for articles related to neurotoxicity of PAHs, as shown in Figure 1. The wildcard * was applied to improve the recovery of articles indexed with inflectional forms of the search terms in the databases. The returned articles were downloaded in the comma-separated (CSV Excel), BibTeX (bib) and Tab-delimited (Win, UTF-8) formats for pre-processing and further analyses.

### 2.2. Data Analysis

The duplication of articles and associated variables were normalized using the ScientoPy package [32] and bibliometrix package [33] using python and R environments. The following parameters were harmonized: spelling anomalies in author details (name and affiliations (institution and country)) and article details (keywords and source). This is to prevent inflationary or deflationary errors in the outcomes of data analysis. R programming environment was used to analyse the trend, descriptive and productivity datasets. The descriptive variables consisted of the yearly production with the mean citations/articles and (co)-author indices (i.e., authors number, articles/author, number of appearances, single- and multi-authored articles, (co-) authors/article and collaboration index). The top 20 active authors, countries and journals were identified according to their productivity using their number of citations and corresponding H-index. The methods of Aria and Cuccurullo [33] and Ekundayo et al. [34] were used to rate the research outputs on the neurotoxicity of PAHs using a co-word analysis of author–keywords regulated to their root forms by the Porter stemming algorithm. The multidimensional metric scaling (MDS) results of the unsupervised k-means clustering were then visualized.

### 2.3. Country Collaboration Mapping of PAH Neurotoxicity Research Landscape

The methods of Aria and Cuccurullo [33] were followed and employed to create a country network concerning articles on PAH neurotoxicity using bibliometrix package in the R environment as a square (adjacency) matrix of countries by publications. The presentation of the network obtained was achieved following the description of Jaccard [35]. The methods of Mao and Zhang [36] were followed to calculate the main network statistics.

## 3. Results

### 3.1. Annual Research Outputs

**Figure 2:**
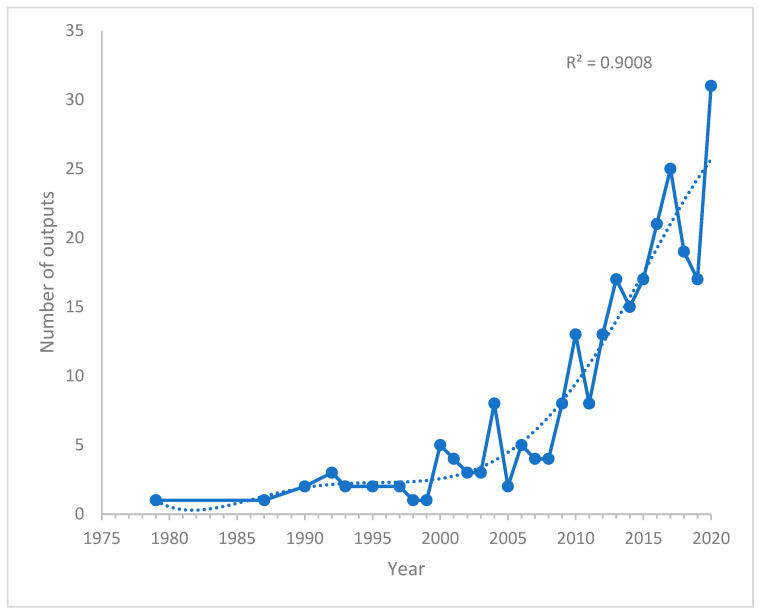
Annual research outputs on PAH neurotoxicity related research.

The annual research outputs related to investigations on PAH neurotoxicity are presented in Figure 2. An increase in research outputs was observed from 1987 to 1992. However, a decrease in outputs was identified between 1992 and 1999. From the year 2000, a rise and fall in research output were observed until the year 2020. Moreover, an increase in research outputs was observed from 2010–2020. Despite the fluctuations in research outputs for 20 years, the trend in Figure 2 revealed a significant increase in research outputs in the last five years (2015–2020) compared to previous years.

### 3.2. Main Information

Table 1 depicts the global mapping of research related to PAH-induced neurotoxicity from 1979–2020 based on the search results obtained from Web of Science and Scopus. The search results showed a total of 338 articles from both databases. Moreover, 80 articles were excluded due to duplications, while 258 articles were used for the study. The articles were published from 129 sources, including 246 journal articles, 4 book chapters, 1 early access article, and 7 proceeding papers. A total of 1192 prolific researchers authored the articles, with about 12 authors of single-authored documents and 1180 authors of multi-authored documents, which means that multiple authors predominantly wrote the documents. The average citations per document were 29.41, and the documents consist of 2970 keywords plus and 851 author’s keywords. The co-authors per document were 6.17, and the collaboration index was 4.82, which revealed a strong collaboration among the researchers who authored these documents.

### 3.3. Analysis of Most Productive Authors

The top 11 most productive authors are listed in Table 1. The search approach revealed Perera F. as the most productive author with 14 outputs in research related to PAH neurotoxicity. Three authors, including Ramesh A., Raugh V. and Tang, produced ten outputs each. The total number of citations of top-cited articles ranged from 63–509. The most cited article was published in Environmental Science Technology in 1993, with a total citation of 18.1 per year. The article published by Avio et al. [37] was the second most cited paper, with 411 citations and about 68.5 citations per year. Allen et al. [38] published the least cited paper, with 69 citations and 17.25 citations per year.

### 3.4. Country Collaboration

**Figure 3:**
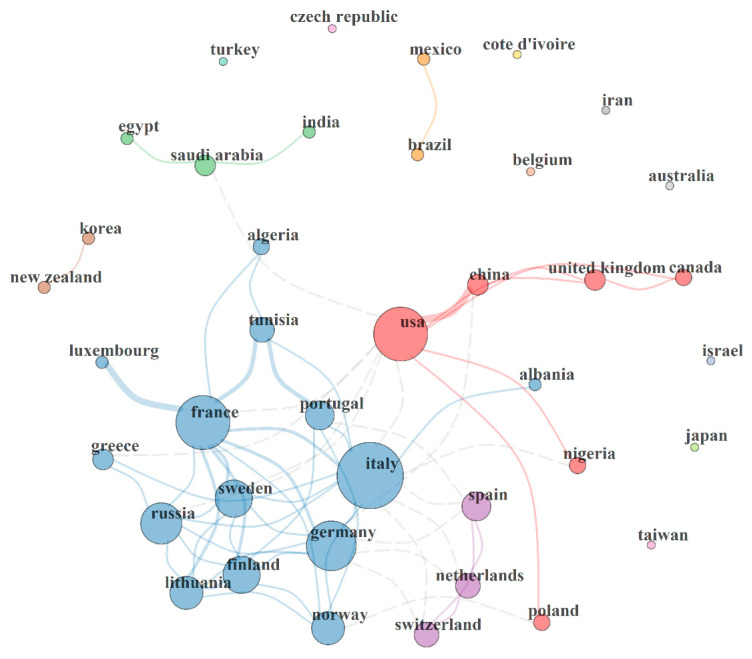
Country collaboration map. Network statistics: size = 39; density = 0.088; transitivity = 0.435; diameter = 4; degree centralization = 0.281; closeness centralization = 0.033; betweenness centralization = 0.164; eigenvector centralization = 0.767; average path length = 2.211.

Figure 3 revealed the existing collaboration network between countries involved in research related to PAH neurotoxicity. Each country is represented by a circle with varying sizes which connotes the total number of publications produced by each country. This means the bigger the circle, the higher the number of outputs. The link or connection between two or more countries has a varying thickness of the connection lines, which connotes the strength of collaboration between different countries. Figure 3 revealed six main collaboration clusters, including two brown and yellow nations, three green and purple nations, six red nations and fourteen blue nations.

The brown and yellow clusters connote collaboration networks between New Zealand and Korea, and Mexico and Brazil respectively. The three-nation green cluster revealed a collaboration network between Egypt, Saudia Arabia and India, while the purple cluster showed a link between Spain, The Netherlands and Switzerland. Furthermore, the six-nation red cluster showed a collaboration network between USA, China, Canada, United Kingdom, Nigeria and Poland. A strong link was observed between USA and China. Moreover, the fourteen-nation blue cluster showed a collaboration link between France, Portugal, Russia, Luxembourg, Sweden, Greece, Italy, Germany, Finland, Lithuania, Norway, Albania, Tunisia and Algeria. Strong collaboration links were observed in the blue cluster, which includes France and Luxembourg, France and Italy, France and Tunisia, Portugal and France, Sweden and Lithuania, and Sweden and France. The observed collaboration networks will strengthen research investigation on PAH neurotoxicity and reveal possible biochemical and pathological mechanisms associated with neurodegeneration and neurological disorders caused by different PAHs. The strong collaboration links observed between different countries will also increase research outputs and bring about new ideas regarding potential therapeutic interventions to mitigate PAH-induced neurotoxicity. Figure 3 also showed the performance statistic of the collaboration network between difference countries which include size = 39; density = 0.088; transitivity = 0.435; diameter = 4; degree centralization = 0.281; closeness centralization = 0.033; betweenness centralization = 0.164; eigenvector centralization = 0.767 and average path length = 2.211.

### 3.5. Thematic Areas

**Figure 4:**
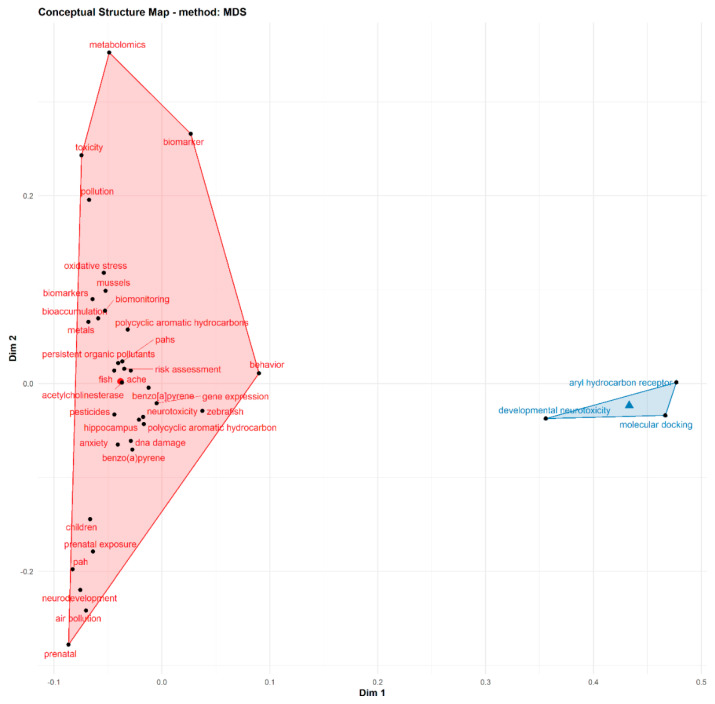
Thematic areas and conceptual landscapes on PAH neurotoxicity research.

Figure 4 depicts the conceptual framework structure and thematic areas in retrieved articles on PAH neurotoxicity. Two major conceptual thematic areas were identified. The first cluster (red) revealed biomonitoring and assessment of health risk of PAH exposure in human and animal models. This cluster depicts the risk assessment, biomonitoring and bioaccumulation of PAHs in different experimental models such as mussels, fish, zebrafish, rats and human models (children and prenatal exposure). Significant compounds/chemicals highlighted in the articles include Benzo[a]pyrene, persistent organic pollutants, pesticides and metals. Furthermore, target biomarkers in experimental models which were investigated in identified articles include oxidative stress, acetylcholinesterase, gene expression, DNA damage, anxiety and behavioural studies, hippocampus and metabolomics. The second cluster revealed developmental neurotoxicity and the structure-activity relationship involving aryl-hydrocarbon receptors as biochemical mechanisms involved in PAH-induced neurotoxicity.

**Figure 5:**
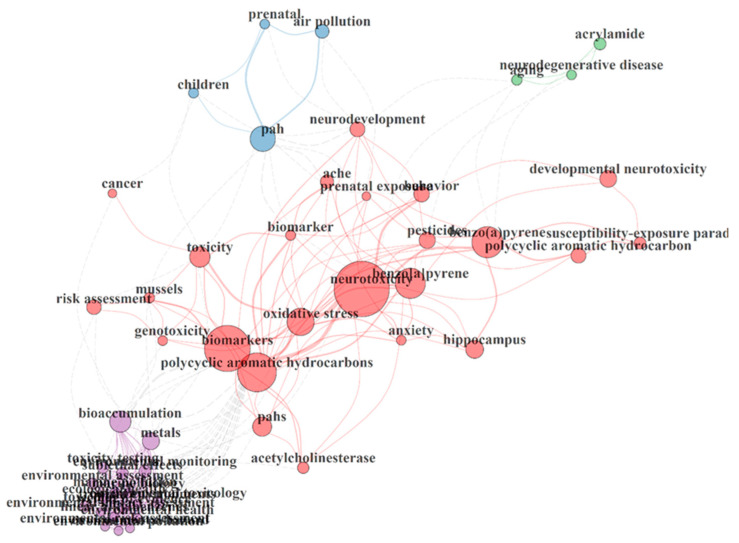
Keyword occurrence map of PAH neurotoxicity research.

The keyword co-occurrence network map linked with research on PAH neurotoxicity is shown in Figure 5. Four different clusters were observed from the analysis of the keywords co-occurrence network obtained from the retrieved articles. The first cluster in purple revealed some keywords from topics that gained attention in PAH neurotoxicity research. This includes bioaccumulation, environmental assessment, environmental monitoring, environmental toxicology, sub-lethal effects, toxicity testing and toxicology. The second cluster in blue revealed related topics on prenatal exposure, children and air pollution. Furthermore, acrylamide, aging and neurodegenerative diseases were observed in the third cluster green, which are consequences of PAH neurotoxicity. The red cluster that revealed the highest number of keywords showed links between major target markers, PAHs and experimental models identified in the databases retrieved. These include oxidative stress, developmental neurotoxicity, acetylcholinesterase, prenatal behaviour, anxiety and genotoxicity.

### 3.6. Corresponding Authors

Table 2 revealed detailed information on corresponding authors from different countries of the world. A total of 72 articles were affiliated to USA, with 64 single country publications (SCPs) and 8 multiple country publications (MCPs). China had 23 articles, with 8 SCPs and 5 MCPs, while France had 18 articles, with 12 SCPs and 6 MCPs. Finland, Korea, Sweden, Tunisia and Poland had 3 articles, with varying SCPs and MCPs.

### 3.7. Country Analysis

The results of the country analysis obtained from the search in Web of Science and Scopus databases are shown in Table 2. USA (2145) was the country with the highest number of citations, followed by Portugal (699) and Canada (643), with average citations of 29.8, 63.5 and 71.4, respectively. Japan (65), Tunisia (64) and Korea (60) had the least citations, with average citations of 32.5, 21.3 and 20, respectively.

### 3.8. Journal Analysis

Some of the most relevant journals that published articles on PAH neurotoxicity are shown in Table 3. *Science of the Total Environment* (16) published more articles than other journals on PAH neurotoxicity related research topics. While *Neurotoxicology* published 13 articles, *Toxicological Sciences*, *Environmental Research*, *Environmental Science and Pollution Research*, and *Aquatic Toxicology* published 11, 9, 9 and 8 articles, respectively, between 1979 and 2020. Altogether, these journals comprise 35.3% of all articles retrieved from our search analysis.

## 4. Discussion

### 4.1. Global Research Output Related to PAH Neurotoxicity

This review is the first systematic assessment of research trends related to neurotoxicity of PAHs from 1979 to 2020. About 258 published articles retrieved from SCOPUS and Web of Science were used for the study. Our findings revealed that research outputs revealing the neurotoxic characteristics of some PAHs was low from 1979 to 2009. However, recognition of PAHs as environmental contaminants with neurotoxic properties increased drastically from 2010 to 2020. Within the last 10 years, an increase in publications related to PAH neurotoxicity was observed in different scientific journals, especially journals such as *Science of the Total Environment*, *Neurotoxicology*, *Toxicological Sciences*, *Environmental Research* and *Environmental Science and Pollution Research*. These were the top five journals that published the highest number of articles that reported PAH neurotoxicity. The exponential increase observed in PAH-induced neurotoxicity related research may be due to an increase in awareness of some PAHs, especially benzo[a]pyrene as neurotoxic compounds. Furthermore, the insight and development of different experimental models, including in vitro and in vivo techniques, may have also triggered the increase in PAH neurotoxicity research outputs in the last ten years.

The inclusive overview of research trends observed in the results also suggests an advancement in research related to neurotoxicity of PAHs in the studied period. These could be seen in the difference between the research outputs produced in 1979–2009 and the last ten years (2010–2020). Only 60 articles were published in 1979–2009. However, the number of articles published in 2010–2020 increased to 198. All the articles published under the studied period were written by 1192 authors across different countries of the world.

These authors used different experimental methods and techniques to determine PAH neurotoxicity, which involved in vitro, in vivo and human trials. According to the articles retrieved from the two databases used in this study, an analysis of studies published in 1979–2009 revealed a focus on identifying PAHs as neurotoxicants. However, the studies were limited to the neurotoxic effects of benzo[a]pyrene in cell lines and animal models. One of the studies revealed that benzo[a]pyrene induced acute neurobehavioural toxicity via suppression of motor activity and antioxidant defense system in rat brains [39]. Jayasekara et al. [40] reported short term exposure to benzo[a]pyrene inhibited monoamine oxidase activity and increased neuronal activity via elevation of biogenic amines (5-hydroxytryptamine, norepinephrine and dopamine) in different brain regions (medulla oblongata, hypothalamus and striatum). Neurotoxic effects of individual PAHs such as naphthalene, pyrene, Chrysene, anthracene, benz[a,h]anthrancene and fluoranthene are scarce. Furthermore, studies on the neurotoxic effects of mixtures and/or combinations of compounds that are PAHs were reported. Andersson et al. [41] reported that intrastriatal and intrahippocampal injections of fractions of exhaust emission containing PAHs caused loss of acetylcholinesterase and tyrosine hydroxylase and induced minimal lesions in the striatum and hippocampal brain regions. One of the major research breakthroughs highlighted between 1979 and 2009 was neurodevelopmental toxicity of PAHs. One of the studies revealed that benzo[a]pyrene disrupted developing neurons, which may cause adverse neurodevelopmental effects via impairment in neurodifferentiation in PC12 cells [42]. Bouayed et al. [43] also observed that benzo[a]pyrene caused neurobiological changes, behavioural disturbances and motor activity problems and was linked to adverse postnatal neurodevelopment and behaviour in mice. Furthermore, McCallister, Maguire, Ramesh, Aimin, Liu, Khoshbouei, Aschner, Ebner and Hood [20] established that prenatal exposure to benzo[a]pyrene might reduce cerebrocortical mRNA expression and induce deficits in cortical neuronal activity in offspring of the progeny.

More advancement in research related to PAH neurotoxicity was observed from research outputs produced between 2010 and 2020. The authors of these articles leveraged reports from previous years to establish more insights into the biochemical mechanisms involved in PAH neurotoxicity in different experimental models, including zebrafish, rats, prenatal and neurodevelopmental models and human trials. The authors also focused on the assessment of neurotoxic effects of combined mixtures of PAHs and other environmental pollutants such as metals, pesticides and polychlorinated biphenyls. One of the studies revealed that combined exposure of benzo[a]pyrene and nicotine showed disruption of cholinergic function, which may affect cognitive performance [44]. Moreover, the results showed that the disruptive effects of nicotine on the neurotransmitters was worsened by exposure to benzo[a]pyrene, which suggests that the combined effect of both chemicals exacerbates the impact of tobacco smoke on the developing brain. Jinzhu et al. [45] also established that co-exposure of aluminium and benzo[a]pyrene synergistically induced apoptosis in rat neuronal cultures.

Furthermore, studies on the therapeutic approach to mitigate PAH-induced neurotoxicity were also considered in the last decade. A study showed that melatonin exhibited neuroprotection against benzo[a]pyrene induced neuronal oxidative stress, neuronal death and autophagy [46]. The study of Mohanty et al. [47] also revealed the role of retinoic acid as a possible therapeutic intervention against benzo[a]pyrene induced neurotoxicity via attenuation of oxidative damage to the brain and mitigation of neurobehavioral and neuromorphological deficits in a zebrafish model.

The result from the annual scientific production and percentage growth rate from 1979 to 2020 was 12.57%. This connotes relatively good progress in research associated with PAH neurotoxicity. It also indicates that PAHs are chemicals of environmental concern and their impact on mental health is critical. This has assisted researchers in identifying possible pathological mechanisms and therapeutic interventions for the prevention of PAH-induced neurotoxicity. The top 20 cited articles with total citations ranging from 76–509 revealed their contributions to fundamental knowledge in research topics associated with PAH neurotoxicity. Some of these studies focused on the effect of PAHs, especially benzo[a]pyrene and anthracene, on acetylcholinesterase activity, antioxidant enzymes and redox imbalance in different experimental models. Other studies also revealed the neurobehavioural toxicity of PAHs and their effect on cognitive function. Most cited studies also showed the consequences of prenatal exposure to PAHs, which may lead to behavioural problems, disruption of white matter, cognitive and attention deficits in late childhood. Some studies also emphasized the vulnerability of fetuses and young children to PAHs and the neurotoxic effect of tobacco smoke. With respect to citations, articles published by authors from USA, Portugal, Canada, France and Italy were the most cited articles and the top five. The total number of citations for articles published by authors from China, Spain, Sweden, the United Kingdom and Australia were below the top five. The average article citation is an indicator of the impact of research outputs produced by the most productive countries. The average citations to the number of published articles of top 10 countries is in the following order: Sweden > Australia > Canada > Portugal > United Kingdom > Poland > France > Japan > Italy > USA. This result revealed that research articles produced by researchers in Sweden had the highest impact on PAH neurotoxicity research and could have served as a reference for other relevant studies. Canada had the highest impact of papers in North America, while Japan had the highest in Asia.

Moreover, authors from USA were more prolific in publishing more articles related to PAH neurotoxicity than other countries such as China, France, Australia and India. These countries were the top five most prolific countries in publishing articles related to PAH neurotoxicity. The high contributions to PAH neurotoxicity research observed from these countries may be linked to the ability to identify the gaps and impact of PAH neurotoxicity in their immediate environment, quality research investigations and output and innovative ideas to prevent and combat the consequences of these organic neurotoxic agents countries on the list of 20 countries involved in the investigation of neurotoxicity of PAHs.

The co-authorship analysis revealed the collaboration network among different countries that investigated the effect of some PAHs on the central nervous system. A strong collaboration link was observed between the USA and China, which could be the reason for the high level of output produced by these countries. Other collaboration links were identified between China and Italy, the United Kingdom and Canada; however, these links were not as strong as the collaboration between USA and China. Apart from Collaborating with China, USA also exhibited moderately strong collaboration with Nigeria, Poland, United Kingdom and Canada, and a weak link with some European countries such as Italy, Sweden, Russia and France. Collaborative networks on research related to PAH neurotoxicity was also observed among some European countries, especially Portugal, France, Sweden, Greece, Finland, Germany, Lithuania, Norway, Spain, Netherlands and Switzerland. However, some of the most robust collaboration links observed are between France and Italy, Luxembourg and France, Germany and Italy, France and Germany, Sweden and France, and Finland and Germany. The solid collaborative networks observed in these countries, especially among European countries and China and USA, could be linked to the awareness and impact of environmental pollutants triggered by industrialization and fuel consumption in different countries and the need for environmental protection. In a study conducted by Edokpayi, Odiyo, Popoola and Msagati [3], the result showed that biomass combustion was the primary source of PAHs while effluents from wastewater treatment plants contributed to the major anthropogenic source of PAHs. Furthermore, Li, Zhou, Jia, Ge, Mei, Sui, Wang, Li, Wang and Wu [6] showed that China’s environmental protection strategies had yielded significant results. However, the economy is growing, industries are developing, fuel consumption is increasing and pollutants in the environment are still high. Hence, the government may need to invest more in environmental protection measures, which will strongly impact research contributions and collaborative networks. No collaboration network was identified among African countries except the link between Algeria and Tunisia. Nigeria also co-authored papers with USA and Italy. Furthermore, two important clusters of collaborative networks were identified. The most productive collaboration network is the blue cluster which consists of 18 countries, most of which are European countries. The second cluster (red) revealed collaborative networks between six countries (USA, China, United Kingdom, Canada, Nigeria and Poland). This collaborative network seems to be productive as most of the articles and citations related to PAH neurotoxicity were accrued to some of the countries in this cluster (USA, China, United Kingdom and Canada). However, some countries that are present at the edge of the map had weak collaborative networks with other nations, which might have affected their research outputs related to PAH neurotoxicity. Some of these countries include Japan, Taiwan, Israel, Australia, Belgium, Iran, Cote d’Ivoire, Czech Republic and Turkey.

### 4.2. Neuropathological Mechanisms of PAHs

PAHs and their metabolites may cross the blood–brain barrier, hence gaining access into the central nervous system, thereby eliciting neurological abormalities including neuronal damage, learning and memory problems impaired neurotransmitter regulation, parasympathetic dysregulation and neurodegeneration. Some of the neuropathological mechanisms of PAHs are highlighted in Table 4 and discussed below.

#### 4.2.1. Effect of PAHs on Antioxidant Defence System

Investigations into the role of oxidative stress in PAH-neurotoxicity is one of the preliminary studies that established the neuropathological mechanism of PAHs. Different experimental models such as zebrafish, neuronal cells and rats were employed to determine how PAHs may induce oxidative damage in the nervous system. However, out of the 16 priority PAHs identified by regulatory bodies, benzo[a]pyrene-induced oxidative stress was reported more than other PAHs in all the articles retrieved. Exposure to 0.4 µg/L of benzo[a]pyrene altered brain antioxidant status via elevation of malondialdehyde and protein carbonylation, reduced glutathione levels and inhibition of catalase, glutathione reductase and glutathione -S- transferase activities in zebrafish [47]. In a rat model exposed to benzo[a]pyrene, alteration of motor activity and behavioural function was observed and attributed to the suppression of antioxidant enzymes (superoxide dismutase and glutathione peroxidase) and elevation of malondialdehyde levels in the striatum and hippocampus [39]. Lin et al. [48] reported the molecular basis of benzo[a]pyrene-induced neurotoxicity during embryogenesis in zebrafish via upregulation of oxidative stress related genes (*sod1*, *sod2* and *cyp1a1*) and increase in malondialdehyde production. Other studies also established that benzo[a]pyrene disrupted brain antioxidant defence systems in vitro and in vivo by triggering lipid peroxidation and inhibiting antioxidant enzymes [46,49,50]. High levels of malondialdehyde and a weak antioxidant defence system have been linked to radical-induced neuronal damage and neurodegeneration [51]. Hence, one of the neuropathological mechanisms of benzo[a]pyrene-induced neurotoxicity in the brain is via induction of oxidative stress, which involves redox imbalance and free radical attack to the neurons.

#### 4.2.2. PAHs and Induction of Apoptosis

Another important neuropathological action of PAHs, especially benzo[a]pyrene, is the induction of apoptosis in different brain regions. Reactive oxygen species formed due to an imbalance of the antioxidant system play a vital role in apoptosis [58,59]. Neuronal apoptosis involves programmed cell death, while autophagy has been identified as programmed cell death II. Autophagy pathways help maintain cell homeostasis as it regulates the removal of wastes, aged cells and damaged organelles and promotes cell survival and energy production. However, disruption of autophagy pathways may lead to cell death. Some of the articles retrieved from the databases showed a link between neuronal apoptosis and autophagy and benzo[a]pyrene-induced neurotoxicity. One of the authors established that benzo[a]pyrene induced neuronal death via disruption of apoptotic markers and autophagy proteins [46]. The study showed that exposure to 75 mg/kg of benzo[a]pyrene upregulated cleaved caspase-3 activity but did not alter the level of pro-caspase 3 and Bcl-2 in the mouse brain. Other studies also showed benzo[a]pyrene-induced neuronal apoptosis via upregulation of apoptotic markers such as p53, Bax, caspase-3, caspase-9 C-myc and Ki67 and downregulation of Bcl-2 proteins in rats hippocampus and cortex [60,61]. The effect of benzo[a]pyrene on autophagy pathways also increased LC3 II/I ratio and Beclin1 protein in mouse brain. Gao, Wu, Wang, Wang and Zuo [53] reported that chronic exposure to benzo[a]pyrene triggered neuronal apoptosis in the telencephalon of zebrafish.

#### 4.2.3. Effect of PAHs on Acetylcholinesterase Activity

The cholinergic system plays a vital role in the nervous system due to its involvement in neurotransmission and memory function. Hence, the disruption of cholinergic function may impair cognitive function. In the cholinergic pathway, acetylcholinesterase regulates acetylcholine levels in the synaptic cleft. Acetylcholinesterase is one of the most studied enzymes as a marker of neurotoxicity, neurite growth, neurodevelopment and synaptic integrity [62]. Acetylcholine is a neurotransmitter that transfers nerve impulses from one neuron to another. Different studies have targeted acetylcholinesterase as a target biomarker for PAH neurotoxicity. The inhibition of acetylcholinesterase activity by these environmental pollutants may disrupt cholinergic function and trigger the accumulation of unbound acetylcholine at the synaptic cleft, leading to hyperstimulation of cholinergic and muscarinic receptors in the nervous system. In an in vitro study carried out by Hauser-Davis et al. [63], naphthalene and its metabolites 2-naphthol, chrysene, phenanthrene, pyrene and 1-OH-pyrene inhibited acetylcholinesterase (AChE) activity in mullet brain homogenates. The study showed that phenanthrene exhibited higher inhibitory effects than chrysene and naphthalene and established that these PAHs might be potent AChE inhibitors. Furthermore, it was observed that 1-naphthol showed a less inhibitory effect compared to its parent compound, naphthalene, which suggests that hydrolyzed products are less toxic than their parent compound. The AChE inhibitory effects of some PAHs have been attributed to the number of aromatic rings in their structure. Kang and Fang [64] established that PAHs with more aromatic rings in their molecular structure exhibited higher inhibitory effects on AChE activity than those with lesser rings. Sediments containing different concentrations of PAHs (pyrene, chrysene, benzo[a]pyrene, benzo[a]anthracene, indenopyrene, benzo[b]fluoranthene, etc.) induced cholinergic dysfunction in mussels via inhibition of acetylcholinesterase and choline acetyltransferase activities [65]. Other studies addressing the effect of PAHs on cholinergic function revealed that these neurotoxic agents, including benzo[a]pyrene and anthracene, induced cholinergic deficit in vivo (in rats and zebrafish) via upregulation of nicotinic acetylcholine receptors, choline acetyltransferase and acetylcholinesterase activities [44,66,67].

#### 4.2.4. PAHs and Developmental Neurotoxicity

Some of the studies retrieved in the search analysis also focused on neurodevelopmental toxicity of PAHs. Most of these studies were published between 2017–2020. The trend of research outputs within this period showed that the attention of most investigators shifted from the evaluation of acetylcholinesterase to neurodevelopmental toxicity. The sudden increase in studies on PAHs and developmental neurotoxicity could be due to the increase in awareness and scientific indications that children and fetuses exposed to environmental pollutants may affect developmental processes and cause adverse effects in the developing brain. The study of Chen et al. [68] and McCallister, Maguire, Ramesh, Aimin, Liu, Khoshbouei, Aschner, Ebner and Hood [20] were the earliest research investigations and most cited articles on neurodevelopmental toxicity of PAHs. Both studies used different experimental approaches to determine the effect of prenatal exposure to PAHs on the nervous system. Prenatal exposure to a low dose of benzo[a]pyrene reduced cerebrocortical expression of glutamatergic NMDA receptor, which may led to cortical neuronal deficits later in the offspring [20]. While the study of Chen, Tang, Jiang, Qi, Cheng, Qiu, Peng and Tu [68] showed that postnatal exposure of neonate Sprague-Dawley pups to 2 mg/kg of benzo[a]pyrene caused behavioural impairment, which may not be noticeable in juveniles but present in childhood and could be long-lasting.

Moreover, in subsequent years, more studies confirmed the results of McCallister, Maguire, Ramesh, Aimin, Liu, Khoshbouei, Aschner, Ebner and Hood [20] and Chen, Tang, Jiang, Qi, Cheng, Qiu, Peng and Tu [68], revealing PAHs’ developmental neurotoxicity using different experimental models. The study of Slotkin, Skavicus, Ko, Levin and Seidler [44] suggested that smoking in pregnant women or fetuses exposed to smoke from cigarettes containing nicotine and benzo[a]pyerene may be harmful and may cause developmental brain problems, disruption of cholinergic and serotonergic systems. In an in vitro PC12 cell line model, Slotkin and Seidler [42] showed that benzo[a]pyrene might trigger adverse neurodevelopmental effects by impairing neurodifferentiation in neuronal cells via an increase in cell numbers and reduction of tyrosine hydroxylase and choline acetyltransferase. Another important mechanism of PAH-induced developmental neurotoxicity reported by two different authors involves targeting cord blood PAH/aromatic DNA adduct, brain-derived neurotrophic factor (BDNF) and long interspersed nuclear elements (*LINE1*) DNA methylation [69,70]. DNA methylation plays a vital role in development, genomic printing and gene transcription [69,71], while BDNF is important for early breurodevelopment, neurological survival and cognitive function [72,73]. Exposure to PAHs triggered a decrease in DNA methylation [74,75]. Moreover, Lee, Kalia, Perera, Herbstman, Li, Nie, Qu, Yu and Tang [69] measured the levels of *LINE1* methylation as a potential mediator between PHA-adduct and neurodevelopmental toxicity. The authors suggested that *LINE1* DNA methylation is a potential molecular indicator for prenatal exposure eurodevelopmentaletal toxicity in children. However, Perera, Phillips, Wang, Roen, Herbstman, Rauh, Wang and Tang [70] suggested that prenatal exposure to air pollutants caused PHA/aromatic DNA adducts which reduced BDNF levels and mental development index at age 2 or 3 years.

#### 4.2.5. Effect of PAHs on Learning, Behaviour and Memory Function

Impairment in learning and memory and behavioural problems are endpoints of the consequences of neurotoxicity and neurodegeneration. Furthermore, a behavioural response is mainly linked to disruptions in neurochemicals nervous systems and neurotoxicity. Some brain regions, especially the temporal lobe structures, including the hippocampus and cortical regions, are associated with memory function and behaviour. A recent neuroimaging study conducted in elderly individuals exposed to PAHs revealed cortical thinning and adverse effects on temporal, parietal, frontal and insular lobes in the brain [76]. A significant decline in verbal learning and memory function was observed in the study and was linked to neurodegeneration due to relatively high exposures to PAHs. The possible mechanism of action of PAHs is linked to activation of inflammatory markers and pathways, which may lead to neuroinflammation and subsequent degeneration of sensitive regions associated with memory. Some of the studies identified in Scopus and Web of Science revealed the link between exposure to some PAHs and memory function, learning and behavioural response. In this study, the most cited article in SCOPUS that focused on learning and memory revealed a unique molecular mechanism of benzo[a]pyrene-induced neurotoxicity. Exposure to benzo[a]pyrene showed learning and cognitive impairment via alterations of some neurotransmitter receptor gene expression in rat hippocampus. This involves downregulation of dopamine receptor gene (Drd1a and Drd2) and upregulation of the α5-GABA receptor. These two receptor genes perform different neurophysiological functions in the brain’s hippocampal region to enhance memory function. While the dopamine receptor genes are involved in integrating spatial memory, recognition memory, temporal retrieval memory function, regulation of acetylcholine release and long term memory retrieval in the hippocampus, a5-GABA receptors mediate cognitive processes, tonic inhibition and attenuation of synaptic plasticity. Disruption of the expression of these receptors will affect neuroactive ligand-receptor interaction associated with learning and memory function.

Another mode of action of benzo[a]pyrene involves alteration of hippocampal N-methyl D-aspartate (NMDA) expression, which may disrupt NMDA-induced ion currents and reduce long-term potentiation leading to impaired memory and learning functions [77]. Furthermore, exposure to benzo[a]pyrene also caused learning and memory dysfunction by inducing oxidative damage in the brain via alterations of antioxidant enzymes and the formation of malondialdehyde [78]. The pathological lesions caused by oxidative stress in the brain’s hippocampal region leads to neurodegeneration and impairs memory function. Molecular mechanism of benzo[a]pyrene-induced learning and memory impairment also involves alteration of oncogenes (Bcl-2, C-myc and Ki-67) and proapoptotic (p-53, Bax and Caspase-3) gene expression in rats’ hippocampal and cortex brain regions [61]. One of the major highlights of studies on prenatal and postnatal exposure to PAHs is that behavioural deficit and memory problems may not be evident at the developmental or juvenile stages but can occur later in childhood and adulthood. This hypothesis was proven in a study conducted by Crepeaux et al. [79], which revealed that exposure to mixtures of PAHs during gestation and breastfeeding season triggered an increase in anxiety-associated behaviours and alteration of metabolic processes linked with memory function and learning. The behavioural deficit and anxiety-like behaviours were linked to impaired locomotor activity and reduced cytochrome C oxidase activity in the amygdala, limbic system, hypothalamus and hippocampus (CA1, CA2 and dentate gyrus), which are associated with cognitive function, fear and identification of emotions. A decrease in cytochrome oxidase activity is linked to hypometabolism and mitochondrial dysfunction, which suggest that redox imbalance and oxidative stress may induce neuronal damage and may trigger the observed neurobehavioural deficits [79,80]. Moreover, the authors emphasized that perinatal exposure to the PAH mixtures showed negative neurological impacts after 40 days of exposure and not at the early stage. This revealed that late consequences might be exhibited during early exposure to PAH mixtures. However, further investigations revealed that prenatal exposure to the same PAH mixtures did not exhibit significant neurobehavioral impairment, as shown by behavioural and cognitive function tests, including determination of cerebral metabolism and histochemical analysis of brain regions associated with cognitive function [81]. The period of exposure to PAH mixtures is very important, and early exposure, especially at development stages, play a major role in inducing neurobehavioral problems and cognitive dysfunction later in adulthood [79,81]. Early postnatal exposure (PND 5–6 days) to benzo[a]pyrene (2 mg/kg) altered locomotor activity after 36 days and became more evident after 69 days exposure [68]. In the same study, early postnatal exposure to benzo[a]pyrene triggered spatial memory deficit later in adulthood.

The study of Sugahara et al. [82] showed the effect of pyrene and phenanthrene on neurodevelopment and behavioural function in an early hatched putterfish larvae experimental model. The behavioural analysis showed that exposure to 100 ppb of pyrene impaired swimming trajectory and reduced optic tectum. Moreover, a higher concentration of phenanthrene (200 ppb) also caused abnormal swimming patterns. The observed uncoordinated swimming patterns and behavioural disturbances were linked to malformed sensory circuits in the midbrain due to inhibition of optic tectum cell proliferation receiving afferent axons and disrupting connections between neurons. Mixtures of ten different PAHs (pyrene, retene, fluoranthene, benzo[a]anthracene, chrysene, naphthalene, acenaphthene, phenanthrene, fluorene and 2-methylnaphthalene) commonly found in surface water were exposed to zebrafish at developmental stages [83]. PAH mixtures caused neurobehavioural deficits in the adult fish by reducing learning capacity and ability to respond to environmental stimuli. Another study involving chronic and long term dietary exposure of three different mixtures of PAHs containing either high, low or intermediate concentrations of PAHs caused behavioural disruptions in zebrafish evident by anxiety-like behaviour [84]. Continuous exposure to fluorene (1, 10 and 100 mg/kg) revealed cerebral levels of parent compounds which contributed to changes in animal behaviour in a rat model of fluorene-induced behavioural toxicity [21]. A clinical study on consistent occupation exposure to PAH showed behavioural toxicity in coal mine workers [85]. Coal miners exposed to high levels of PAH exhibited neurobehavioural deficit, which was revealed by impairment in auditory and visual memory and reduction in the ability to process information

#### 4.2.6. PAHs and Neurodegeneration

Neurodegeneration is a consequence of neuronal injury, and evidence has shown that it contributes to the progression of diseases such as Parkinson’s disease and Alzheimer’s disease [86,87]. Consistent exposure to some environmental pollutants over a long period may affect the nervous system, cause neuronal injury and subsequently neurodegeneration [88,89]. Though the effect of PAHs on memory function, behavioural toxicity and learning capacity has been established in different experimental models. However, studies involving experimental investigations on PAHs and pathological mechanisms (mitochondrial dysfunction, neuroinflammation, beta-amyloid aggregation, tau phosphorylation, formation of α-synuclein) associated with Parkinson’s disease and Alzheimer’s disease are still very few. Moreover, there are indications that cumulative exposure to PAHs may trigger neurodegenerative disorders. In a study conducted by Valand et al. [90], long-term exposure to disease exhaust emission caused mild histopathological alterations in the rat’s brain’s frontal cortex and hippocampal region. Some of the PAHs identified in the diesel exhaust include anthracene, fluorene, phenanthrene, fluoranthene, naphthalene, pyrene, chrysene and benzo[a]anthracene. Mild neuronal damage, degeneration and minimal neuronophagy were observed in the frontal cortex and hippocampus. The observed minimal histopathological alteration may accumulate and increase the disruption of neurological processes, including neurotransmission in the central nervous system. An experimental investigation involving chronic exposure to benzo[a]pyrene also revealed histopathological changes associated with PD and AD. A loss of dopaminergic neurons, reduction of dopamine levels and accumulation of beta-amyloid were observed in the brain of zebrafish exposed to benzo[a]pyrene [53]. These neuropathological alterations are hallmarks of PD and AD, which progressively lead to neurodegeneration. Furthermore, some genes associated with PD (*DAT* and *LRRK2*) and AD (*APPb*, *PSEN1* and *PSEN2*) were also altered in the brain. This is an indication that benzo[a]pyrene may trigger AD and PD-like neuropathological characteristics.

Furthermore, alteration of neuroinflammatory responses is a significant risk factor for neurodegeneration. PAHs have been identified as activators or suppressors of inflammatory responses [91]. An exposure to air pollution particulate matter containing PAHs showed the activation of IFNγ and NF-κB in rat brain. These neuroinflammatory factors have been implicated in pathological processes (formation of neurofibrillary tangles, accumulation of neuritic plaques, production of inflammatory cytokines and induction of cholinergic degeneration) leading to neurodegeneration [92].

### 4.3. Therapeutic Strategies against PAH-Induced Neurotoxicity

Several studies have established the neurotoxicity of PAHs, including their combinatory effects with other organic pollutants to induce neurodegeneration, behavioural problems and cognitive dysfunction. Possible therapeutic intervention to mitigate the neuropathological problems caused by PAHs will be a potential research topic to explore. There are indications that some therapeutic agents at low doses may induce some adaptive responses that may confer neuroprotection against toxic stimuli at different cellular conditions caused by envirnmental pollutants and other chemicals [93,94]. Seven articles related to therapeutic intervention against PAH neurotoxicity were identified in Scopus and Web of Science, revealing limited reports on this topic. Most of the articles identified focused on neuroprotection and were limited to benzo[a]pyrene-induced model of experiment. Possible pharmacological mechanisms to mitigate neuropathological events caused by other PAHs such as fluorene, benz[a]anthracene and anthracene are yet to be investigated. One of the studies revealed that co-supplementation with retinoic acid attenuated PAH neurotoxicity via mitigation of anxiolytic-like behaviour and oxidative damage [47]. Neuroprotective potentials of retinoic acid against benzo[a]pyrene-induced neurotoxicity were associated with different biochemical mechanisms, including reduction of malondialdehyde and protein carbonyl production, activation of antioxidant enzymes, inhibition of neuronal pyknosis and prevention of behavioural alteration. A recent study revealed that melatonin also exhibited neuroprotection against benzo[a]pyrene-induced neurotoxicity via reduction of apoptotic markers and inhibition of neuronal apoptosis and autophagy [46]. The report of Saha et al. [95] also showed that a medicinal plant, *Bacopa monnieri*, may provide beneficial effects against PAH-induced neurological deficit. *B. monnieri* exhibited neuroprotective effects against benzo[a]pyrene-induced neurotoxicity via its anti-senescence and anti-apoptotic effects by activating mitochondrial autophagy in the mitochondrial of astrocytes. Butylated hydroxyanisole (BHA), a potent antioxidant, also showed neuroprotection and may mitigate the consequences of sub-chronic exposure to benzo[a]pyrene via attenuation of oxidative damage and modulation of purinergic enzymes (Na^+^/K^+^-ATPase and Ca^2+^/Mg^2+^-ATPase), which may improve memory and learning processes [50].

## 5. Conclusions and Future Perspectives

This study revealed that, between 1979 and 2020, 258 papers related to the neurotoxicity of polycyclic aromatic hydrocarbons were published in reputable journals indexed in Web of Science and Scopus. These papers were identified using specific keywords (Polycyclic aromatic hydrocarbon and neurotoxicity). The leading countries in this field of research are USA, China, France, Portugal and Canada. Research productivity was rated based on the number of publications and citations. In the last ten years, the global scientific output in research relating to PAH neurotoxicity has focused on neurodegeneration, cholinergic function, neurodevelopment, behavioural studies, oxidative stress, neuroprotection and therapeutic intervention. Furthermore, most studies investigated these research topics using different models, including zebrafish, *Caenorhabditis elegans* and neuronal cell lines. Few studies investigated PAH neurotoxicity in preclinical trials and human studies. Furthermore, most studies also focused on the neurotoxic effects of benzo[a]pyrene; hence, more studies on other priority PAHs and their mixtures are required. Studies on the biochemical and molecular mechanisms of PAH neurotoxicity and their effects on biomarkers (mitochondrial dysfunction, beta-amyloid aggregation, tau phosphorylation and dopaminergic system) associated with Alzheimer’s disease and Parkinson’s disease are also recommended to be made.

## Figures and Tables

**Table 1:** Main information and most productive authors of articles on PAH neurotoxicity.

Variable	Count/Rate	Authors	Articles
Documents	258	Perera F.	14
Sources (journals, books, etc.)	129	Ramesh A.	10
Keyword plus (ID)	2970	Raugh V.	10
Author’s keywords (DE)	851	Tang D.	10
Period	1979–2020	Hood D.	9
Average citations per document	29.41	Schroeder H.	8
Authors	1192	Das S.	7
Author’s appearances	1592	Budzinski H.	6
Authors of single-authored documents	12	Herbstman J.	6
Authors of multi-authored documents	1180	Rychen G.	6
Single authored documents	13	Wang S.	6
Documents per author	0.216		
Authors per document	4.62		
Co-authors per document	6.17		
Collaboration index	4.82		
Documents types			
Article	246		
Article, book chapter	4		
Article, early access	1		
Article, proceedings paper	7		

**Table 2:** Countries of corresponding authors and total citations per country on articles related to PAH neurotoxicity published between 1979 and 2020.

	Productivity Based on Articles Published						Productivity Based on Citations			
Rank	Country	Articles	Freq	SCP	MCP	MCP Ratio	Rank	Country	Total Citations	Average Total Citations
1	USA	72	0.30769	64	8	0.1111	1	USA	2145	29.7
2	China	23	0.09829	18	5	0.2174	2	Portugal	699	63.5
3	France	18	0.07692	12	6	0.3333	3	Canada	643	71.4
4	Italy	14	0.05983	9	5	0.3571	4	France	597	33.2
5	India	12	0.05128	11	1	0.0833	5	Italy	453	32.4
6	Portugal	11	0.04701	5	6	0.5455	6	China	295	12.8
7	Brazil	10	0.04274	9	1	0.1	7	Spain	269	26.9
7	Spain	10	0.04274	9	1	0.1	8	Sweden	264	88
8	Canada	9	0.03846	8	1	0.1111	9	United Kingdom	228	45.6
9	Germany	7	0.02991	7	0	0	10	Australia	175	87.5
9	Norway	7	0.02991	5	2	0.2857		Germany	163	23.3
10	United Kingdom	5	0.02137	5	0	0	10	India	163	13.6
11	Finland	3	0.01282	1	2	0.6667	11	Poland	118	39.3
11	Korea	3	0.01282	2	1	0.3333	12	Belgium	108	54
11	Poland	3	0.01282	2	1	0.3333	13	Finland	87	29
11	Sweden	3	0.01282	3	0	0	14	Norway	86	12.3
11	Tunisia	3	0.01282	0	3	1	15	Brazil	75	7.5
							16	Japan	65	32.5
							19	Tunisia	64	21.3
							20	Korea	60	20

MCP: multiple country publications; SCP: single country publications.

**Table 3:** Most relevant sources on PAH neurotoxicity research from 1979–2020.

Sources	Articles
*Science Of The Total Environment*	16
*Neurotoxicology*	13
*Toxicological Sciences*	11
*Environmental Research*	9
*Environmental Science and Pollution Research*	9
*Aquatic Toxicology*	8
*Marine Pollution Bulletin*	7
*Environment International*	6
*Plos One*	6
*Toxicology Letters*	6

**Table 4:** Effect of some PAHs on cognitive and behavioural function in different experimental models.

Authors	Study Duration/Design/Model of Experiment	Sample Size	PAHs Investigated	Dosage/Route of Administration	Main Outcome Indicator	Neuropathological Mechanism
Das and Patri [49]	male Wistar pups (adolescent rats)/30 days	54	benzo[a]pyrene	0.2 µg/kg BW/Intracisternal	1, 2, 3, 4	- Induction of neurodegeneration via elevation of MDA levels, low GPx activity and over expression neuropeptide Y in hippocampus and hypothalamus; - increase in serotonin levels after treatment with benzo[a]pyrene; - induction anxiolytic-like behavioural response.
Liang, Tang, Duan, Cheng, Luo, Cao and Tu [50]	adult male rats/90 days	96	benzo[a]pyrene	2 mg/kg BW/Intragastric	1, 5, 6, 7	- Hippocampal oxidative damage; - increase ATPase activity; - impaired learning and memory function.
Lin, Wu, Hu, Pai, Chen and Wang [48]	wild type and transgenic zebrafish (embryos)/20–22 h		benzo[a]pyrene	10 and 20 µM	1, 5, 8	- Oxidative stress induced developmental neurotoxicity; - reduction of hypoxia-inducible factors signalling and downregu; - lation of gene expression;
Patel et al. [52]	male Wistar pups (5 day old)/30 days	18	benzo[a]pyrene	0.2 µg/kg BW Intracisternal	1, 2, 4, 7, 9, 10, 11, 12, 13	- impaired antioxidant signalling; - alteration in hippocampal cytomorphometry; - pyknotic cell death; - altered behavioural response; - impaired differential migration of neurons.
Saunders, Das, Ramesh, Shockley and Mukherjee [39]	rats/2–96 h	50	benzo[a]pyrene	25–200 mg/kg BW	1, 5, 9, 13	- Redox imbalance; - altered behavioural response.
Mohanty, Das and Patri [47]	adult zebrafish/7 days		benzo[a]pyrene	1–4 nM	1, 9, 10, 11, 15, 16	- Oxidative stress-induced neurodegeneration; - neurobehavioral and neuromorphological alterations.
Gao et al. [53]	zebrafish/230 days		benzo[a]pyrene	0.05–53.93 nmol/L	15, 17, 18, 19	- Altered neurochemical and neurobehavioural features; - loss of dopaminergic neurons; - impaired locomotor and cognitive ability; - neuronal death.
Mehri, Barangi, Zamiri and Karimi [46]	male Razi mice/28 days	30	benzo[a]pyrene	75 mg/kg	1, 12, 20, 21, 22, 23	- Induction of oxidative stress, apoptosis and autophagy.
Xia et al. [54]	rats/13 weeks		benzo[a]pyrene	2.5 and 6.25 mg/kg BW	1, 5, 6, 24, 25,2 6, 27, 2829	- Impaired behavioural performance; - disruption of neurotransmitters; - cholinergic and monoaminergic dysfunction; - impaired memory function and learning; - oxidative stress-induced neuronal damage.
Slotkin, Skavicus, Ko, Levin and Seidler [44]	sprague dawley rats/		benzo[a]pyrene	30 mg/kg/day	26, 29	- Impaired cholinergic and serotonergic systems.
Gauthier et al. [55]	amphipod—*Hyalella azteca*		phenanthrene	195 µg L^−1^	24, 30	- AChE Inhibition; - ROS production; - severe behavioural impairment.
Saunders et al. [56]	F-344 rats		fluoranthene	100–400 mg/kg		- Reduced motor activity; - neuromuscular weakness; - decreased reactions to sensory stimuli; - autonomic deficits.
He et al. [57]	rock fish (*Sebastiscus marmoratus)*		pyrene	0.5 and 50 nmol/L	24, 26, 31, 32	- neural pattern defects; - decreased synaptic structural plasticity; - suppression of neural outgrowth; - impaired CaMKII and CREB expression.

1—malondialdehyde, 2—glutathione peroxidase; 3—neuropeptide Y; 4—elevated plus maze; 5—Superoxide dismutase activity; 6—Morris water maze; 7—ATPase activity; 8—hypoxia inducible factors; 9—Catalase; 10—glutathione reductase; 11—glutathione-S transferase; 12—glutathione levels; 13—motor coordination; 15—novel tank diving test; 16—protein carbonylation; 17—beta-amyloid peptide (Aβ-42); 18—Dopamine; 19—PSEN1 and 2; 20—Sirt1; 21—LC3II/I; 22—Beclin I; 23—Caspase-3; 24—acetylcholinesterase; 25—choline acetyltransferase; 26—acetylcholine; 27—monoamine; 28—adrenaline; 29—5-hydroxytryptamine; 30—reactive oxygen species; 31—CaMKII; 32—CREB.

## Data Availability

Data available on request from the corresponding author.
